# Psychological Clinical Science: Meeting the Challenge of Public Mental Health

**DOI:** 10.32872/cpe.12067

**Published:** 2024-04-26

**Authors:** Richard J. McNally

**Affiliations:** 1 Department of Psychology, Harvard University, Cambridge, MA, USA; Department of Psychology, University of Trier, Trier, Germany

**Keywords:** clinical science, Psychological Clinical Science Accreditation System, PCSAS, public mental health

## Abstract

The purpose of this article is to provide a brief overview of how clinical psychology evolved in the United States as a prelude to discussing the emergence of psychological clinical science in the closing years of the 20th century. Despite the growth of clinical psychology, mental disorders remain highly prevalent, compelling us to envision new ways to deliver services in an effective but efficient manner. Topics include the dissemination gap, the affordable access gap, and the Psychological Clinical Science Accreditation System (PCSAS). Examples of novel methods for addressing the problem of public mental health in the 21st century are discussed. Finally, I close by considering the potential relevance of our experience in America for European clinical psychology.

## Highlights

- The history of clinical psychology in America is traced.
- The clinical science movement emerged from struggles with the APA’s accreditation system.
- The development of the Psychological Clinical Science Accreditation System (PCSAS) is described.
- Can the clinical science model flourish in Europe?

## The Origins of Clinical Psychology in America

The founder of experimental psychology, Wilhelm Wundt, was an immensely productive man. He was the author or co-author of 503 publications (Simonton, 2002, p. 37) and the supervisor of 186 Ph.D. dissertations, including those of 16 Americans who had traveled to the University of Leipzig to study under his direction (Benjamin et al., 1992). One of these students, Lightner Witmer, coined the term *clinical psychology* (Witmer, 1907/1996). Like many of Wundt’s American students, Witmer was eager to apply the new science of psychology to practical problems. Upon his return from Germany, he established the first psychological clinic in 1896. Based at the University of Pennsylvania, the clinic aimed to help children who were struggling in school, had difficulties with attention or memory, or who exhibited disruptive behavior. Witmer and his assistants worked closely with physicians, social workers, and schoolteachers (Witmer, 1909).

Meanwhile, three faculty members in the precursor to Harvard University’s Department of Psychology – William James, Morton Prince, and Hugo Münsterberg – were engaging in psychotherapy, often involving hypnosis, to treat adult patients with psychoneuroses (Taylor, 2000). Each of them had an M.D. and Münsterberg also had a Ph.D. earned under Wundt’s mentorship. However, President Charles Eliot of Harvard advised Münsterberg to cease “the hypnotic treatment of women” after one of Münsterberg’s patients smuggled a pistol into a therapy session and threatened to shoot him, causing a bit of a scandal.^1^1I thank Ludy T. Benjamin, Jr. for providing me with a copy of President Eliot’s letter to Professor Münsterberg, dated April 30, 1909.

Despite these early beginnings, psychotherapy was not the main role for clinical psychologists during the first half of the 20^th^ century (Benjamin, 2005). Indeed, psychiatrists treated patients with psychosis, and neurologists, such as Freud in Austria and Münsterberg in the United States, treated patients with neuroses. Although some self-described clinical psychologists began practicing psychotherapy, physicians vigorously and effectively opposed them, claiming that psychotherapy was solely the province of medicine. The upshot was that clinical psychologists were largely confined to developing, administering, and interpreting what another American mentee of Wundt, James McKeen Cattell, called *mental tests.* Tests of cognitive ability, personality, and psychopathology (e.g., Minnesota Multiphasic Personality Inventory) figured prominently in the careers of clinical psychologists who often worked closely with educators, the military, and psychiatry.

## The Golden Age of Clinical Psychology

World War II changed everything. Despite psychiatric screening of military inductees designed to eliminate the psychologically vulnerable, psychiatric battle casualties were very common and some never recovered. Approximately 60% of the men receiving medical treatment from the Veterans Administration (VA) in the late 1940s were suffering from the psychiatric consequences of warfare (Levenson, 2017). There were far too few psychiatrists to treat the tsunami of cases, and so the VA asked the American Psychological Association (APA) to establish a formal curriculum to train clinical psychologists capable of delivering psychotherapy to troubled veterans. Leaders of the field convened at the University of Colorado and formulated an educational and training curriculum known as the Boulder or scientist-practitioner model of clinical psychology (Committee on Training in Clinical Psychology of the American Psychological Association, 1947). Clinical psychologists were to be hybrids. They conducted research worthy of the scholarly Ph.D. and they received clinical training, including an internship, thereby qualifying them to join psychiatrists as psychotherapists.

In the late 1940s, the APA assumed the responsibility of evaluating clinical psychology Ph.D. programs and giving its stamp of approval for those that met its accreditation criteria. The federal government poured vast amounts of money into the VA and university departments of psychology to support the training of clinical psychologists. The number of accredited clinical psychology programs grew, and graduates joined the ranks of VA practitioners, others became professors, and very many others commenced lucrative careers in the private practice of psychotherapy. In the United States today, 44.7% of clinical psychologists are in private practice, 17% practice in hospitals, and 11% work in universities.

The profession of clinical psychology grew immensely in the following decades, but so did dissatisfaction with the APA’s scientist-practitioner model. A group of 14 prominent practitioners who called themselves “the Dirty Dozen” transformed clinical psychology in America (Wright & Cummings, 2001). The reference to dirt in their self-applied moniker denotes their willingness to engage in political lobbying on behalf of their profession as well as “all sorts of psychologically unseemly acts” (Wright, 2001, p. 2). They wrote a revelatory book describing how they gained control over the APA and used it as a vehicle for “professionalizing” clinical psychology. Their volume is a self-congratulatory tale of triumph over both psychiatry and over the science-oriented, academic clinical psychologists whose attitudes regarding practice, they argued, ranged from benign neglect to hostile contempt. The Dirty Dozen believed programs accredited by APA were biased toward research at the expense of preparing graduates for clinical practice – the chief career goal for most graduates. Utterly clueless about the intensely competitive healthcare marketplace, academic clinicians, they said, were wholly inept at lobbying Congress in defense of the professional and economic interests of clinical psychologists in private practice who were struggling to compete against social workers and psychiatrists for healthcare dollars in the 1980s as managed care in the health insurance industry began to curb reimbursement for mental health.

The politically astute Dirty Dozen and their allies in private practice secured control of state psychological associations and eventually the power structure of APA itself. Four of them were elected president of APA: Theodore H. Blau in 1977, Nicholas A. Cummings in 1979, Max Siegel in 1983, and Jack G. Wiggins in 1992. Among their achievements was ensuring that clinical psychologists could obtain acceptable reimbursement for their services from insurance companies governed by managed care.

Rejecting the scientist/practitioner model, some Dirty Dozen members established proprietary professional schools of clinical psychology awarding the Doctor of Psychology degree (i.e., the Psy.D.) which does not require a research-based dissertation. Following a conference held in Vail, Colorado in 1973, their practitioner-scholar model of clinical training was recognized by the APA as an accreditation-eligible approach to clinical training.

Yet there are ironies to the Dirty Dozen’s approach to professionalizing clinical psychology (McNally, 2003). In the early 20^th^ century, physicians professionalized medicine by *strengthening* the connection between practice and science (Starr, 1982, pp. 112-127), whereas the Dirty Dozen strive to do the opposite. Unfortunately, their gambit will likely undermine the professional status of our field. As sociologists emphasize (e.g., Freidson, 2001, pp. 152-176), a profession must possess epistemic authority to survive. Practitioners acquire prestige when they possess specialized knowledge and expertise unavailable to those outside the profession. A clinical psychology increasingly divorced from science will cease to command the allegiance of clients, Congress, or society at large.

Moreover, as medicine professionalized by bolstering its scientific base in the early 20^th^ century, many free-standing proprietary medical schools vanished (Starr, 1982, p. 118). They folded because they lacked funds for laboratories, libraries, and the technology that were available to university-based medical schools, such as those at Johns Hopkins and Harvard. Ironically, members of the Dirty Dozen have been among the most enthusiastic supporters of proprietary professional schools.

## The Emergence of Clinical Science

The Dirty Dozen repudiated the scientist-practitioner model because they believed that it overemphasized often-irrelevant science at the expense of training for a successful career in the private practice of psychotherapy. Yet another group of clinical psychologists were also dissatisfied with the model, but for precisely the opposite reason. Their views were canonically captured in McFall’s (1991) essay, “Manifesto for a Science of Clinical Psychology” (See also, McFall, 2000). McFall argued that the scientist-practitioner model implied that a clinical psychologist can be either a scientist or a practitioner, thereby suggesting that scientific reasoning, empirical principles, and evidence-based assessment and treatment are not necessarily relevant for a practitioner of psychotherapy. He argued that the Ph.D. first and foremost confirms the psychologist as a scientist regardless of whether he or she works in a lab or in a clinic.

McFall emphasized that scientific clinical psychology is – or should be – the *only* clinical psychology. Just as patients rightly expect that their cardiologists, oncologists, and internists will always base their practice on the best available science, patients of clinical psychologists have every right to expect the same. Unfortunately, in the years since McFall’s manifesto, our field has remained cluttered with popular interventions whose efficacy remains empirically untested (e.g., Bessel van der Kolk’s ‘the body keeps the score’ approach to treating trauma; McNally, 2023); tested, but ineffective (e.g., psychological debriefing for trauma; McNally et al., 2003); or downright harmful (For a review, see Lilienfeld, 2007).

McFall’s call to arms resonated with many Directors of Clinical Training (DCTs) of the scientifically strongest clinical psychology Ph.D. programs and clinical internship programs, resulting in the founding of the Academy of Psychological Clinical Science in 1994 (Benjamin, 2005). Academy members struggled within APA to strengthen the role of science in the training of psychotherapists and managed to convince APA to recognize the *clinical science* as the third accreditable training model. As of this writing, the APA has accredited 108 Psy.D. and 256 Ph.D. programs in clinical psychology, and 68 of these Ph.D. programs are members of the Academy of Psychological Clinical Science. Hence, of the three models of training – scientist-practitioner, practitioner-scholar, and clinical scientist – clinical scientist programs are in the minority.

Continued frustration with the APA accreditation system motivated the Academy to development an alternative, science-based clinical psychology accreditation system to rival that of the APA. A vote of Academy members overwhelmingly supported and authorized this system in 2007, named the Psychological Clinical Science Accreditation System (PCSAS). The founding Executive Director was Richard M. McFall.

The goal of PCSAS is to foster excellent, science-centered education and training in university programs granting the Ph.D. in clinical psychology and to advance the knowledge base for disseminating and delivering the safest, most cost-effective psychological health services to the public. Note that *clinical science* is transtheoretical. Although many programs favor a cognitive-behavioral approach to training and treatment, such an orientation is independent of the empirical, scienced-based focus of PCSAS.

Also, diverse meta-theoretical perspectives are compatible with the clinical science approach including categorical diagnostic (e.g., American Psychiatric Association, 2013; Haeffel et al., 2022), dimensional (e.g., Kotov et al., 2017; Kotov et al., 2021), and network analytic (e.g., Borsboom, 2017; McNally, 2021) ones. Hence, the guiding questions regarding an intervention are: “Does it work?” and if so, “How do we know?”

One major difference between APA and PCSAS accreditation procedures concerns input versus output. That is, the APA has a checklist of course and content coverage essential for accreditation, whereas PCSAS emphasizes the *outcomes* of training. Are graduates of clinical science programs functioning as clinical scientists? For example, the criteria for classifying a graduate as functioning as a *clinical scientist* in the Department of Psychology at Harvard University is as follows:

1. The graduate of our clinical science program is generating new knowledge via research. Evidence of this may be employment as a postdoctoral fellow, faculty member at a college or university, or scientist at a research facility or hospital affiliated with a medical school.
2. Their position involves the widespread dissemination of clinical science research. This may be accomplished through scholarly publications, conference presentations, teaching of clinical science related courses, or research supervision. Any publications are expected to go beyond work done solely in graduate school.
3. Leadership role in a clinical setting involving program development, new initiatives in training or assessment, or public policy work where the graduate is clearly using clinical science skills. Simple application of evidence-based assessment and treatment in a clinical setting (e.g., a VA) is not sufficient.

For accreditation, PCSAS requires that at least 50% of a program’s graduates qualify as clinical scientists.

As of this writing, PCSAS has accredited 46 Ph.D. programs in clinical psychology at universities in the United States and Canada. These are among the finest programs. *U.S. News & World Report* ranks the top clinical psychology programs in America, and 40 of the top 50 programs are accredited by PCSAS as are all 20 of the top-ranked programs.

## The Challenge of Public Mental Health

The Academy sponsored the *Summit on Clinical Science Training* at Washington University in St. Louis on May 4-5, 2023. The purpose was to address pressing issues concerning our field. Most participants were DCTs or representatives of other relevant stakeholders (e.g., National Institute of Mental Health). The videos of the major talks and summaries of the intensive breakout discussion groups are now available online as are the lists of participants for each of these groups.^2^2The videos summarizing the content of the Summit on Clinical Science Training can be viewed here: https://www.acadpsychclinicalscience.org/summitproceedings.html. The topics and members of the workgroups can be viewed here: https://drive.google.com/file/d/1_GZOpkqmL1iWhMczyRhw0-Ia7YJhCvmJ/view?usp=sharing.

The Summit covered a range of topics including how best to select Ph.D. students for clinical science training, concerns about how to streamline course curricula, mentoring models, ensuring the mental health of our Ph.D. students^3^3My research group has devised a brief, scalable workshop for teaching emotion regulation skills to help Ph.D. students manage stress and counteract burnout, modified to accommodate the stressors characteristic of diverse departments (e.g., physics, economics, psychology, philosophy, etc.). Our pre-post data are favorable (Bernstein et al., 2021; Bernstein et al., 2023)., underrepresentation of racial minorities in clinical science,^4^4For the past several years, Harvard’s Department of Psychology have offered a free, intensive weekend workshop delivered nationally via Zoom providing mentoring guidance for college students keen on obtaining a Ph.D. in psychology (including clinical science) at universities throughout the country. The enrollees are from throughout the United States, and are members of underrepresented minority groups, first-generation college students, and others who are unlikely to acquire the tacit knowledge about the educational and other experiences enabling applicants to gain admission to graduate school. Our aim is to level the playing field by transmitting key knowledge, which is readily available to upper-middle class undergraduates, to potential applicants who are otherwise unlikely to be acquainted with it. The link to our program is here: https://psychology.fas.harvard.edu/pprep and occupational opportunities for psychological clinical scientists outside academia (e.g., in government, in think tanks). Some of these issues have been thoughtfully discussed by Gee et al. (2022).

However, the chief challenge was how to improve public mental health. Despite the emergence of evidence-based treatment protocols for many common mental disorders (e.g., Barlow, 2021), epidemiologic data indicate that we failing to move the needle regarding their incidence and prevalence (Insel, 2022). Why?

One explanation is a failure of clinical scientists to disseminate treatments established as efficacious in randomized controlled trials (RCTs). The assumption is that clinical training programs, including clinical practicum and internship sites, are failing to teach these interventions to their clinical psychology trainees.

A related explanation is there is insufficient time for students to master all the evidence-based treatment programs that have been confirmed as efficacious in RCTs. David H. Barlow and his team have addressed this problem by developing and confirming the efficacy of their Unified Protocol for treating the often-comorbid syndromes of depression, panic disorder, and so forth (Barlow et al., 2017). This transdiagnostic approach targets problems that often co-occur in different disorders (e.g., avoidance behavior, emotion regulation problems), thereby obviating the need to master many disorder-specific, evidence-based treatment manuals.

Although the dissemination gap is surely a problem, there is also a treatment access gap, at least in the United States (McNally & McNally, 2016). Because practitioners who are expert in evidence-based psychotherapy possess a relatively rare set of skills, their services are in high demand. Accordingly, they can set their fees as a function of the market and need not accept insurance. Relatively affluent patients can afford to write a check to pay for such expert care, but less affluent patients who rely on their health insurance are out of luck. This problem is less common in European countries with comprehensive health insurance coverage.

As Kazdin and Blase (2011) emphasized, the mental health needs in the United States far exceeds the capacity of clinical psychologists trained to deliver face-to-face interventions over the course of several months of weekly 50-minute sessions. The clinical psychologist David M. Clark and the behavioral economist Richard Layard joined forces to solve this problem in England. They developed a remarkable program entitled Improving Access to Psychological Therapies^5^5The program has been renamed the *NHS Talking Therapies for Anxiety and Depression.* (IAPT; Clark, 2018).

Clark and Layard lobbied Labor and the Tories, respectively, making the case that we now have evidenced-based CBT treatments that can ameliorate the suffering of people struggling with depression and anxiety disorders, enabling them to recover and rejoin the work force of productive English citizens. Moreover, Layard calculated, by enabling these patients to return to the workforce as tax-paying, productive citizens who no longer require financial support for psychiatric disability, the program would pay for itself.

By 2018, the senior clinical psychologists had trained over 10,500 new *nondoctoral* therapists to deliver CBT protocols for depression and anxiety disorders. Data were collected for each session to enable progress to be tracked. When frontline therapists encountered difficulties, senior doctoral clinicians were available to provide supervisory guidance. The IAPT program treats more than 560,000 patients per year, and about 50% recover and two-thirds of the remaining patients experience worthwhile progress. The data tracking and feedback mechanisms built into the computerized database enable fine-tuning of clinical practice. Indeed, the effectiveness of the therapeutic interventions has thereby improved over the years since the program was launched in 2008.

Clark and Layard’s remarkable achievements were built on the preexisting National Health Service (NHS). In effect, they made the NHS both more effective and efficient by mandating evidence-based treatment and tracking progress via standardized systematic data collection. I suspect that Clark and Layard’s counterparts in European countries with comprehensive national health coverage could replicate these positive results.

Unfortunately, it would be challenging to accomplish this throughout the United States without a nationwide healthcare system. However, Bradley C. Riemann, Ph.D., has established a conceptually similar program for treatment of OCD in Oconomowoc, Wisconsin at the nonprofit Rogers Behavioral Health System. Riemann, an expert in the behavioral treatment of OCD established inpatient and outpatient services for OCD 28 years ago, and then established an intensive training program for individuals with a B.A. or B.S degree in psychology to conduct intensive *in vivo* exposure and response prevention under the supervision of senior Ph.D. clinical psychologists. Frontline therapists “shadow” expert clinicians conducting behavior therapy and must read a considerable amount of scientific literature on the psychopathology and treatment of OCD, including passing examinations on the material they must master. They, in effect, become highly expert in a narrow area of specialization. Like Clark and Layard, Riemann has a standard ongoing, computerized assessment of OCD, depression, and related symptoms.

Working with expert colleagues throughout the United States, Riemann has now established similar programs at 20 other hospital sites. Strikingly, these paraprofessional therapists for OCD are just as effective as Ph.D. clinicians who are expert in the behavioral treatment of OCD. The upshot is that the number of patients receiving state-of-the-art therapy has vastly increased. In a talk he gave in Paris at the International Convention of Psychological Science (Riemann, 2019), he presented data showing that the percentage of patients receiving intensive treatment increased by 168% and that was when he had established “only” eight additional program in addition to his original one in Wisconsin.

What are the prospects of others replicating Riemann’s achievements elsewhere in America? The essential ingredients appear to be a nonprofit facility keen to offer disorder-specific, efficacious psychological therapy for a relatively common mental disorder (e.g., bulimia nervosa, panic disorder, non-melancholic major depression). When Riemann launched his program, there were only a handful of facilities in the country providing intensive exposure and response prevention for OCD despite its prevalence being much greater than most specialists surmised.

Another possibility for improving public mental health is expansion of training in Barlow’s Unified Protocol. Given its wide applicability, it has the potential of transforming the practice of scientific clinical psychology, especially among “generalist” practitioners.

Barack Obama’s Affordable Care Act (“Obamacare”) was a significant step toward enhancing access to health services in the United States. Further advances, including those specific to mental health, will require considerable political efforts. Although some politicians regard spending on mental health as merely a cost, it is truly an investment in the future. Early detection and efficacious treatment of mental health problems saves money in the long run. Unfortunately, steps that successfully prevent disasters in the distant future seldom seize the attention of politicians preoccupied with the immediate future.

## Conclusion

The most important question, especially for an author writing for *Clinical Psychology in Europe,* is how well favorable trends in America generalize to countries in Europe. I am grateful for two anonymous peer reviewers whose comments partly mitigated my ignorance of the European scene.

Clinical psychology Ph.D. programs in the USA integrate coursework, research, and clinical assessment and treatment practica within departmental clinics or in affiliated clinics (e.g., specialized practica in treating certain disorders in clinics at Harvard Medical School teaching hospitals). Ph.D. programs with a clinical science orientation confine practica to evidence-based sites as well as involve more research activity than do programs with a scientist-practitioner focus. Accredited full-time, 12-month clinical internships complete the requirements for the Ph.D. The best internship sites are those with a compatible clinical science orientation. The course load, the clinical work, and sheer amount of research and writing that students do in a clinical science program hones their time and task management skills, enabling then to flourish despite the heavy workload. The entire process including the internship takes about 6-7 years. Apparently, in some European countries, students complete a research-oriented Ph.D. and then apply to a diversity of clinical training sites to do receiving supervised clinical training in various schools of therapy.

I was surprised to learn how influential psychoanalytic psychotherapy remains in Italy and Germany as well as in France. Psychoanalysts and apparently humanistic psychotherapists vigorously oppose policies confining public spending to mental health services having a solid evidential basis. Moreover, I learned, that when clinical psychologists address the public through the media, they are usually psychoanalytic practitioners, and seldom clinical scientific ones. This is strikingly different from America where mainstream journalists from the *New York Times,* the *Washington Post*, the *Wall Street Journal*, *National Public Radio,* CNN, MSNBC, and other prestigious outlets almost invariably rely on clinical scientists for comment. Apparently, we have a much better chance of influencing public opinion than do many of our continental European counterparts. Alas, dissemination of “pseudoscientific” therapies abounds on social media (e.g., YouTube videos), and thus has a popular platform.

Another route to educating the public other than through the mainstream media is through public policy think tanks concerned with health care. This option was favorably discussed at the St. Louis *Summit on Clinical Science Training* as a career option for clinical scientists who do not plan to work in clinical or academic settings.

Clark and Layard’s program in England is unquestionably the most important clinical science success story in public mental health. By making the case for evidence-based clinical psychology to both the political Left and political Right, they succeeded in gaining bipartisan support for incorporating clinical science into the NHS. The patchwork character of mental health services in the United States poses serious challenges to replicating the English program here. However, I suspect that the public health systems in European countries may be far more amenable of translating Clark and Layard’s system to their respective nations especially as it is just as cost effective as it is efficacious.

In conclusion, clinical science has made major strides in developing treatments for mental disorders, established as efficacious by randomized controlled trials. Yet the need for mental health services is immense. The challenges we face are daunting but are beginning to inspire innovative ways of reaching more people as never before. Ultimately, the prospects for progress are bright if we continue to base our efforts on the best science available.

## References

[r1] American Psychiatric Association. (2013). *Diagnostic and statistical manual of mental disorders* (5th ed.). American Psychiatric Publishing.

[r2] Barlow, D. H. (2021). *Clinical handbook of psychological disorders: A step-by-step treatment manual.* Guilford Press.

[r3] Barlow, D. H., Farchione, T. J., Bullis, J. R., Gallagher, M. W., Murray-Latin, H., Sauer-Zavala, S., Bentley, K. H., Thompson-Hollands, J., Conklin, L. R., Boswell, J. F., Ametai, A., Carl, J. R., Boettcher, H. T., & Cassiello-Robbins, C. (2017). The Unified Protocol for Transdiagnostic Treatment of Emotional Disorders compared with diagnosis-specific protocols for anxiety disorders: A randomized clinical trial. JAMA Psychiatry, 74(9), 875–884. 10.1001/jamapsychiatry.2017.2164 28768327 PMC5710228

[r4] Benjamin, L. T., Jr. (2005). A history of clinical psychology as a profession in America (and a glimpse at its future). Annual Review of Clinical Psychology, 1, 1–30. 10.1146/annurev.clinpsy.1.102803.143758 17716080

[r5] Benjamin, L. T., Jr., Durkin, M., Link, M., Vestal, M., & Acord, J. (1992). Wundt’s American doctoral students. American Psychologist, 47(2), 123–131. 10.1037/0003-066X.47.2.123

[r6] Bernstein, E. E., LeBlanc, N. J., Bentley, K. H., Barreira, P. J., & McNally, R. J. (2021). A single-session workshop to enhance emotional awareness and emotion regulation for graduate students: A pilot study. Cognitive and Behavioral Practice, 28(3), 393–409. 10.1016/j.cbpra.2020.09.008

[r7] Bernstein, E. E., Shingleton, R. M., Finch, E. F., LeBlanc, N. J., Bentley, K. H., Barreira, P., & McNally, R. J. (2023). *A roadmap to address stress in graduate students: How to develop a student-led single session evidence-based intervention*. Manuscript submitted for publication. 10.1080/07448481.2023.2299427 38227928

[r8] Borsboom, D. (2017). A network theory of mental disorders. World Psychiatry: Official Journal of the World Psychiatric Association (WPA), 16(1), 5–13. 10.1002/wps.20375 28127906 PMC5269502

[r9] Clark, D. M. (2018). Realizing the mass public benefit of evidence-based psychological therapies: The IAPT program. Annual Review of Clinical Psychology, 14, 159–183. 10.1146/annurev-clinpsy-050817-084833 29350997 PMC5942544

[r10] Committee on Training in Clinical Psychology of the American Psychological Association. (1947). Recommended graduate training program in clinical psychology. American Psychologist, 2(12), 539–558. 10.1037/h0058236 18917473

[r11] Freidson, P. (2001). *Professionalism: The third logic.* University of Chicago Press.

[r12] Gee, D. G., DeYoung, K. A., McLaughlin, K. A., Tillman, R. M., Barch, D. M., Forbes, E. E., Krueger, R. F., Strauman, T. J., Weierich, M. R., & Shackman, A. J. (2022). Training the next generation of clinical scientists: A data-driven call to action. Annual Review of Clinical Psychology, 18, 43–70. 10.1146/annurev-clinpsy-081219-092500 35216523 PMC9086080

[r13] Haeffel, G. J., Jeronimus, B. F., Fisher, A. J., Kaiser, B. N., Weaver, L. J., Vargas, I., Goodson, J. T., Soyster, P. D., & Lu, W. (2022). The Hierarchical Taxonomy of Psychopathology (HiTOP) is not an improvement over the *DSM.* Clinical Psychological Science, 10(2), 285–290. 10.1177/21677026211068873 36299281 PMC9596130

[r14] Insel, T. (2022). *Healing: Our path from mental illness to mental health.* Penguin Press.

[r15] Kazdin, A. E., & Blase, S. L. (2011). Rebooting psychotherapy research and practice to reduce the burden of mental illness. Perspectives on Psychological Science, 6(1), 21–37. 10.1177/1745691610393527 26162113

[r16] Kotov, R., Krueger, R. F., Watson, D., Achenbach, T. M., Althoff, R. R., Bagby, R. M., Brown, T. A., Carpenter, W. T., Caspi, A., Clark, L. A., Eaton, N. R., Forbes, M. K., Forbush, K. T., Goldberg, D., Hasin, D., Hyman, S. E., Ivanova, M. Y., Lynam, D. R., Markon, K., . . . Zimmerman, M. (2017). The Hierarchical Taxonomy of Psychopathology (HiTOP): A dimensional alternative to traditional nosologies. Journal of Abnormal Psychology, 126(4), 454–477. 10.1037/abn0000258 28333488

[r17] Kotov, R., Krueger, R. F., Watson, D., Cicero, D. C., Conway, C. C., DeYoung, C. G., Eaton, N. R., Forbes, M. K., Hallquist, M. N., Latzman, R. D., Mullins-Sweatt, S. N., Ruggero, C. J., Simms, L. J., Waldman, I. D., Waszczuk, M. A., & Wright, C. G. (2021). The Hierarchical Taxonomy of Psychopathology (HiTOP): A quantitative nosology based on consensus of evidence. Annual Review of Clinical Psychology, 17, 83–108. 10.1146/annurev-clinpsy-081219-093304 33577350

[r18] Levenson, R. W. (2017). Clinical psychology training: Accreditation and beyond. Annual Review of Clinical Psychology, 13, 1–22. 10.1146/annurev-clinpsy-021815-093559 28482690

[r19] Lilienfeld, S. O. (2007). Psychological treatments that cause harm. Perspectives on Psychological Science, 2(1), 53–70. 10.1111/j.1745-6916.2007.00029.x 26151919

[r20] McFall, R. M. (1991). Manifesto for a science of clinical psychology. Clinical Psychologist, 44(6), 75–88.

[r21] McFall, R. M. (2000). Elaborate reflections on a simple manifesto. Applied & Preventive Psychology, 9(1), 5–21. 10.1016/S0962-1849(05)80035-6

[r22] McNally, D. M., & McNally, R. J. (2016). The affordable access gap: CBT’s hidden dissemination problem. Behavior Therapist, 39(1), 21–22.

[r23] McNally, R. J. (2003). The deeds of the Dirty Dozen [Review of the book *The practice of psychology: The battle for professionalism,* by R. H. Wright & N. A. Cummings (Eds.)]. The Scientific Review of Mental Health Practice, 2(1), 72–73.

[r24] McNally, R. J. (2021). Network analysis of psychopathology: Controversies and challenges. Annual Review of Clinical Psychology, 17, 31–53. 10.1146/annurev-clinpsy-081219-092850 33228401

[r25] McNally, R. J. (2023). The return of repression? Evidence from cognitive psychology. Topics in Cognitive Science. Advance online publication. 10.1111/tops.12638 36630259

[r26] McNally, R. J., Bryant, R. A., & Ehlers, A. (2003). Does early psychological intervention promote recovery from posttraumatic stress? Psychological Science in the Public Interest, 4(2), 45–79. 10.1111/1529-1006.01421 26151755

[r27] Riemann, B. C. (2019, March 8-10). Dissemination of a standard training and treatment protocol for OCD in a multi-state behavioral health system: An implementation science model. In R. J. McNally (Chair), *Obsessive-compulsive disorder: Breakthroughs in treatment and theory* [Symposium]. International Convention of Psychological Science, Paris, France.

[r28] Simonton, D. K. (2002). *Great psychologists and their times: Scientific insights into psychology’s history.* American Psychological Association.

[r29] Starr, P. (1982). *The social transformation of medicine.* Basic Books.

[r30] Taylor, E. (2000). Psychotherapeutics and the problematic origins of clinical psychology in America. American Psychologist, 55(9), 1029–1033. 10.1037/0003-066X.55.9.1029 11036704

[r31] Witmer, L. (1996). Clinical psychology. American Psychologist*,* 51(3), 248-251. [Reprinted from The Psychological Clinic, 1, 1-9]. (Original work published 1907) 10.1037/0003-066X.51.3.24828909380

[r32] Witmer, L. (1909). The study and treatment of retardation: A field of applied psychology. Psychological Bulletin, 6(4), 121–126. 10.1037/h0072263

[r33] Wright, R. H. (2001). The rise of professionalism within American psychology and how it came to be: A brief history of the Dirty Dozen. In R. H. Wright & N. A. Cummings (Eds.), *The practice of psychology: The battle for professionalism* (pp. 1-69). Tucker and Theisen.

[r34] Wright, R. H., & Cummings, N. A. (Eds.). (2001). *The practice of psychology: The battle for professionalism*. Tucker and Theisen.

